# Stakeholders’ engagement for improved health outcomes: a research brief to design a tool for better communication and participation

**DOI:** 10.3389/fpubh.2025.1536753

**Published:** 2025-03-26

**Authors:** Giuseppe Pellegrini, Chiara Lovati

**Affiliations:** Observa Science in Society, Vicenza, Italy

**Keywords:** stakeholder engagement, health communication, health technology design, inclusive healthcare, responsible innovation

## Abstract

Technological progress in healthcare is creating a complexity of novelties, from new roles and challenges, to new concerns about inclusivity, equity and data privacy. Communication among healthcare actors becomes a very important factor for adaptation and allows for the improvement of medical research and treatment. Active patient involvement and stakeholder engagement in health research become essential to better information and diagnostic management and communication in a framework of ever-expanding resources defined by the rise of artificial intelligence and other technologies. At the intersection of healthcare, technology and citizenship, the EU-funded Hereditary project plans to facilitate trough Health Social Laboratories (HSL) a multi-level dialog between stakeholders, improving its health outcomes while accounting for the challenges and risks of communication in participatory approaches. This study aims to understand the main issues to address in developing an effective stakeholder collaborative relationship with a focus on communication in a technology-driven context such as the Hereditary project and its health information integration processes. It specifically describes the findings of a literature review, an exploratory context analysis carried out through interviews with 9 key informants from four research partner locations (four interviewees in Italy, two in the Netherlands, one in Spain, and two in the United States), and the transformation of these findings into a prospective laboratory methodology. Our approach aims to highlight the importance of including diverse perspectives in shaping healthcare communication innovation. Through a participative model, researchers can navigate complex ethical and practical healthcare communication challenges more effectively, and foster solutions that are in alignment with stakeholders’ needs.

## 1 Introduction

In recent years, techno-scientific advances and the introduction of increasingly powerful computational tools have radically transformed the healthcare domain (1). Progress in biotechnology, the increased use of electronic health records (EHRs), the advent of “Big Data” and the emergence of artificial intelligence in clinical practice, are changing the way healthcare is delivered, managed, and conceptualized (2). Information and Communication Technologies (ICTs) evolution enables providers to gather huge amounts of data, providing new opportunities and application scenarios (3) that attract high levels of investment and financial support (4). With that, new worries about data privacy, security, and management arise (3).

Progress has added a new layer of complexity to relationships between clinicians, patients and associations (5), making new interaction and communication approaches necessary. Personalized and participatory approaches to medicine (6) are needed, and essential questions about how to best manage health communication and stakeholders’ interactions have to be answered.

Health information needs to be communicated in a relevant, reliable, and accurate way, despite its growing complexity (2): where new diagnostic imaging techniques in clinical contexts are offering complex visualizations that necessitate a specific framework of knowledge for their interpretation, understanding patients’ needs and appropriate health communication strategies becomes of utmost importance (7, 8).

The European Centre for Disease Prevention and Control has defined health communication as “*the study and use of communication strategies to inform and influence individual and community decisions that enhance health”* and has recognized it as a vital element for the improvement of both personal and public health (9). Effective communication between patients, caregivers and healthcare practitioners is critical for the achievement of quality care and to improve access to healthcare and health outcomes. Furthermore, effective health communication is critical in building and reinforcing health literacy, which can empower patients to better navigate healthcare services (10).

Health literacy – a set of individual transferable skills that can aid in reaching greater independence in health decision-making – can help individuals to assess the reliability of health information, and enable them to gain greater control over life events and situations (11).

These connections show the relevance of interaction processes between healthcare actors, clinicians and patients. In addition, studies suggest mechanisms of dialog and active participation of the various subjects involved in health processes can initiate innovation paths (12), and benefit research efforts. Participatory research approaches and the accounting of stakeholders’ needs into research processes have been increasingly recognized as potentially beneficial in several studies (13). Stakeholder engagement in a certain context, it has been noted, can improve the relevance of research, stakeholder trust, and research adoption, create value and knowledge, and enhance both the short-and long-term relevance of clinical research efforts (14, 15). The Hereditary project, funded under the Horizon Europe program, undertook the task of planning a stakeholder engagement model, Health Social Laboratory (HSL), to involve patients, citizens, and domain experts to co-design the project architecture through opportunities for discussions, comparisons, and feedbacks, thus improving its health communication and collaboration efforts through participatory approaches.

The project focuses on multimodal data integration and federated learning in the context of neurodegenerative diseases and gut-brain interplay. It plans to improve information accessibility, comprehensibility and actionability, in order to foster a society that is informed, engaged, and empowered in making informed health choices.

In this context, the following article will provide information about the research efforts taken to design the above stakeholder engagement model to help improve research adherence to stakeholders’ needs and address communication challenges related to the technology-driven Hereditary project context.

We focused on two research questions (RQs).

*RQ1*. What are the main challenges to develop an effective stakeholder collaborative relationship in the health information communication process?

*RQ2*. What are the key communication and technology issues to address for the design of Health Social Laboratories that are specific to the Hereditary project?

In this respect, the research will include an exploratory context analysis aimed at assessing the state of partner networks’ relationships and communication patterns, and an analysis of the main issues to be addressed in the design of new collaborative stakeholder engagement models aimed at improving health communication strategies.

The remainder of this paper is organized as follows. Section 2 describes the research design, data collection and analysis. Section 3 presents the major results of the study. Section 4 explores how the study results are translated into the Health Social Laboratories implementation. Finally, Section 5 presents the conclusions and research answers.

## 2 Methods and analysis

The first step to the building of the Health Social Laboratory (HSL) model included a literature review and comparative analysis of the topic of participatory research and stakeholder engagement to uncover details about the potential of stakeholder collaboration and answer RQ1. The review included identifying, selecting, and evaluating relevant academic sources, and comparatively analyzing them to identify patterns and discrepancies in the approaches and conclusions of the studies, providing a basis for our model design robustness. To begin, a search was conducted across multiple academic databases using keywords such as “participatory health research” and “stakeholder engagement.” In addition to these keywords, the topics of *health*, *technology* and *communication* were prioritized to explore the types of outcomes related to stakeholder interactions in contexts similar to ours.

Only peer-reviewed journal articles, conference papers, and book chapters in English were considered for review. The remaining studies were then assessed for relevance and key data were extracted and synthesized to identify common themes, trends, and gaps in the literature.

This approach allowed us to identify a diverse range of strategies aimed at improved stakeholder collaboration, enhanced health knowledge sharing, and more effective decision-making within the health research context. In this perspective, it was assessed that the HSLs should enable seamless sharing of information across different systems to improve care coordination. Finally, they should favor communication technologies respect cultural norms and align with institutional practices.

The second step in the design of HSLs involved the Hereditary project partner institutions, in order to study their technological, relational and communicative environments. The chosen approach consisted in semi-structured interviews with clinicians and researchers from Hereditary partner institutes. A total of 9 Hereditary collaborators were chosen based on their role within the project and experience in healthcare and/or the health and technology research field. Participants belonged to either universities, hospitals or research centers located in Italy (4), Netherlands (2), Spain (1) and United States (2). These institutes will participate in the realization of HSLs during the project. The selection of the interviewees considered the following criteria: variety of skills (researchers, clinicians, computer scientists) to have a varied information space focused on the different expertise; variety of contexts from the point of view of the relationships between stakeholders; representation of all five case studies foreseen by the project.

Interviewees were invited to participate in individual or group semi-structured interviews aimed at assessing their institutes: *stakeholder network*; *clinical or research activity processes*; *technology and research structure*; *dissemination, communication patterns and efforts*. These four topics influenced the interview guide and questions. The interviews were conducted between March and May 2024, each one lasted between 45 and 120 min, allowing both flexibility and a structured approach. Open-ended questions were employed in order to promote detailed responses and allow participants the chance to fully express their opinions and experiences. Where necessary, follow-up questions were posed to elucidate or elaborate on particular issues.

Interviews were audio-recorded with participants’ consent and subsequently transcribed verbatim. Then, they were subjected to a thematic analysis to identify recurring patterns, themes and insights. Codes were generated to represent key ideas which were then discussed and summarized by the research team. The work was carried out in collaboration by a senior and a junior researcher with a background in sociology and adequate training. In particular they collaborated throughout every stage of the thematic analysis process, including data familiarization, coding, themes generation, review and definition, and finally, reporting. The approach adopted was in line with Byrne’s (16) worked example to reflexive thematic analysis based on Braun and Clarke’s (17) approach, as illustrated in their 2019 commentary on the latter.

A total of 149 codes were identified, and mapped to 23 themes, under the overarching interview topic categories of stakeholders and relationships, clinical activity, technology and research, communication.

This combination of a literature review and interview thematic analysis enabled us tailor the general findings from the literature to our specific area of interest, effectively allowing to answer our research questions.

## 3 Results

As per RQ1, the literature review was aimed at assessing the main issues to address in the development of an effective collaborative stakeholder engagement model to improve health communication processes. The main findings included the following details.

Described as *“referring to the aims, activities, and impacts of stakeholder relations in a moral, strategic, and/or pragmatic manner”* (18), the practice of stakeholder engagement has started to gain popularity at the beginning of the 2000s, particularly in business and society research.

Stakeholder engagement looks to involve “*individuals, organizations or communities that have a direct interest in the process and outcomes of a project, research or policy endeavor*” (19) in a series of specific activities. These activities can vary depending both on the stakeholders’ skills levels and attributes, and on the expertise of the researchers leading the studies (15). These considerations were very important for our interview campaign as we found ourselves dealing with different types of skills that were not always well coordinated with each other.

Stakeholder engagement activities might include activities concerning the co-creation of research questions and methods, data collection, project management, online collaboration, training, and results dissemination. Levels of engagement can range from consultation, to collaboration in partnerships with researchers, to stakeholder-directed projects (13). From this perspective, the interviews allowed us to identify the different levels of involvement of the stakeholders of the five case studies.

Stakeholder collaboration in health information communication processes has the potential to address key challenges concerning, for example, the management of the fast-growing knowledge base, the implications of complex health results and their relative priority to patients, or the administration and privacy, security, and confidentiality of personal health data (20). Stakeholders feedback in health research can be applied to both designs and processes, and can help expand the understanding of the consequences of using emerging technology in clinical practice (21).

The review has highlighted some issues to address in developing a good stakeholder engagement model in health research (22, 23). Practically speaking, some commonly reported challenges in processes such as HSLs included time scarcity on the stakeholder side, or on the facilitating team side, in the identification of suitable representatives to engage in the activities, or a lack of skills, especially due to training or background, in the research or facilitating team involved (13). In addition, the communication between stakeholder can include power asymmetries and diverse communicative needs (24), together with the lack of clarity in the objectives to be achieved. On a more theoretical note, as argued by Wilkinson et al. (25), designers of participatory processes should take steps to avoid resulting in tokenistic or toxic engagement models. Tokenism comes into play with “check-box” types of participation, lacking meaningfulness and/or applicability. This should be counteracted by encouraging broad, inclusive, early and sustained engagement, and valuing stakeholders’ insights regarding the implementation of meaningful participation. The term “toxic” is used to describe the potential for health sector engagement practices to put pressure on already-existing conflictual social relationships within stakeholder groups, as well as the potential for stigma, strain, or other negative effects. In order to address this, participants should be encouraged to express their concerns rather than blindly complying with a predetermined set of rules. Finally, the authors recommend avoiding one-size-fits-all approaches and customizing the participative methodology based on context-specific traits, risks, and concerns (25).

As per RQ2, the analysis of the interviews revealed several key themes related to the participants’ experiences and perspectives on stakeholders, clinical research activity processes, technology and health communication. The last two themes, in particular, provide insights into the key communication and technology challenges that are going to influence the design and content of (HSLs).

On a general note, the interviews allowed to ascertain a high level of multidisciplinarity characterizing the project partners’ institutions, both in terms of expertise and research activities, and varied levels of public engagement and outreach efforts. The identified relationship networks comprised regional, national, and international systems, including patients’ associations, international research groups, and policymaking bodies and governmental organizations. The group of representatives who had experience with diagnostics uncertainty and patients’ needs provided some insights into the different elements of clinical practice and research processes. On the relationship between symptoms and treatment success, versus treatment resistance, it was noted how factors like patients’ psychophysical well-being and bodily autonomy all account for quality of life when dealing with serious illnesses (26).

More relevant to the design of HSLs, on communication and technology interactions, the analysis of the interviews allowed to group communication aims described by the interviewees into technical and societal aims. The first category included communication activities on science, technology, research, or diagnostics, while the second included communication activities for sensibilization, dissemination, or impact. Interviewees reinforced the need for nuanced health communication with patients and/or their relatives. Given the different neurodegenerative diseases considered (Amyotrophic Lateral Sclerosis, Parkinson’s, Multiple Sclerosis), specific information must be given to patients. They underlined challenging elements ranging from demographics to knowledge accessibility issues. It was noted how, for instance, cultural diversity calls for the intercultural adaptation of the messages, or how the varying degrees of skills in risk understanding and critical thinking require the modulation of certain aspects of the communication, such as its complexity or technicality (26). For example, interviewee n. 5 said:

“*If you really need to explain the technology under the hood, then the challenge is maybe to, to use simple words, to make it understandable*”;

while interviewee n. 9 said:

“*I think you have to go for very, very key concept. Very little, like a seed concept in a nutshell when you talk to lay public. Otherwise, it does not, it does not help because people do not understand it*.”

On data and technology use in healthcare that permeates the activity of all partner institutions, the teams were able to provide useful insights into its relationship with clinical and research activity. As interviewee n. 6 said:

“*Data has become a central part of the research that we do*.”

Indeed, technology and data allow partners to conduct research in many different areas, but analysis of the interviews also highlighted how they require specific expertise, such as data management skills, knowledge of data protection normative and legal frameworks, which are not necessarily easy to attain.

Furthermore, as suggested by interviewee n. 7:

“*There’s definitely mixed perceptions from patients about how AI should or should not be used for their own care*.”

In fact, when using data technology in the relationship between clinician or researchers and patient or citizen, some ethical concerns arise. Interviewee n.7 added:

*“There have been questions in the field about the ethics of using patient data for something that’s making somebody else richer, without them getting any benefit.”*

And furthermore:

*“We assume that everybody knows that, that this is a chat bot and a machine talking to you, but half the people did not … What does that mean? When we, when we are starting to thinking, think about deploying chat bots. […] how should we think about it? […] They’re not, I mean, […] the informed consent does not work in the sense that they do not realize that they are not talking to a human being.”*

Implementation of data technology in healthcare can raise worries regarding informed consent, data sharing opt-in, opt-out options, withdrawal rights; on the positive side, however, some interviewees also reported they had managed to raise awareness on the societal value of data and shed light on the potentialities of open access data research through direct dissemination approaches.

This analysis of the Hereditary context proved to be an effective method to identify the key health communication and technology challenges to address in the design of Health Social Laboratories, while also grasping the peculiarities and complexity of the different project partner institutes where HSLs will be realized in the future.

Combining the literature study and the interview analyses, we identified key elements at the intersection of participatory research, technology, and health communication that should be considered in the design of HSLs.

## 4 Implementing health social laboratories

On a theoretical note, the HSL model is based on a Whole Health System approach considering the entire health ecosystem, including patients, providers, institutions, policies, and social determinants. Communication strategies focus on:

- System-Wide Coordination: ensuring that all stakeholders, from patients to policymakers, are aligned on goals and messages;
- Integrated Care Models: using shared platforms to deliver consistent and accurate health information;
- Population Health Focus: addressing communication disparities by tailoring outreach to underserved communities. This approach examines the interaction between people, technologies, and organizational structures in health communication developing tools (e.g., patient portals, apps) that enhance two-way communication while being accessible and user-friendly.

Among the elements mentioned, of particular importance for the realization of the HSLs was to foster a climate of trust to avoid forms of cognitive pressure while respecting the different operational and regulatory priorities. The project partners involved, in fact, have a network of stakeholders organized differently and the relationships between researchers, clinicians and patients are structured with varied intensities. In some cases, there are strong links between all the actors and in others weaker links.

In this sense, HSLs required careful consideration of the following steps in the phase preceding their realization.

- Stakeholder mapping: identify key players and their roles within the health system is one of the main study actions (27) to be able to develop a correct design of the HSLs.
- Transparent information flow: promoting trust by ensuring open, honest, and timely updates to encourage a progressive involvement of stakeholders, allowing them to be prepared for the HSLs.
- Feedback mechanisms: regularly collect information from stakeholders for the purpose of making the HSL protocol.

Given these premises, a framework can be outlined to define the relational principles, the actors who will be appropriately involved and the relationship with the political and healthcare system.

This way, the HSL method will allow real empowerment of the actors involved: for patients, associations and caregivers the possibility of knowing and contributing to the diagnosis and treatment process; clinicians and researcher to improve communication methods and researchers to understand suggestions and recommendations coming from the clinical activities and patients. The indications that emerge from the HSL will have consequences in the definition of healthcare practices (protocols, guidelines, etc.) which will have to be taken into consideration for the development of adequate policies. Information mechanisms, for example, influence the methods of access to treatment and the availability of interventions.

**Figure 1:**
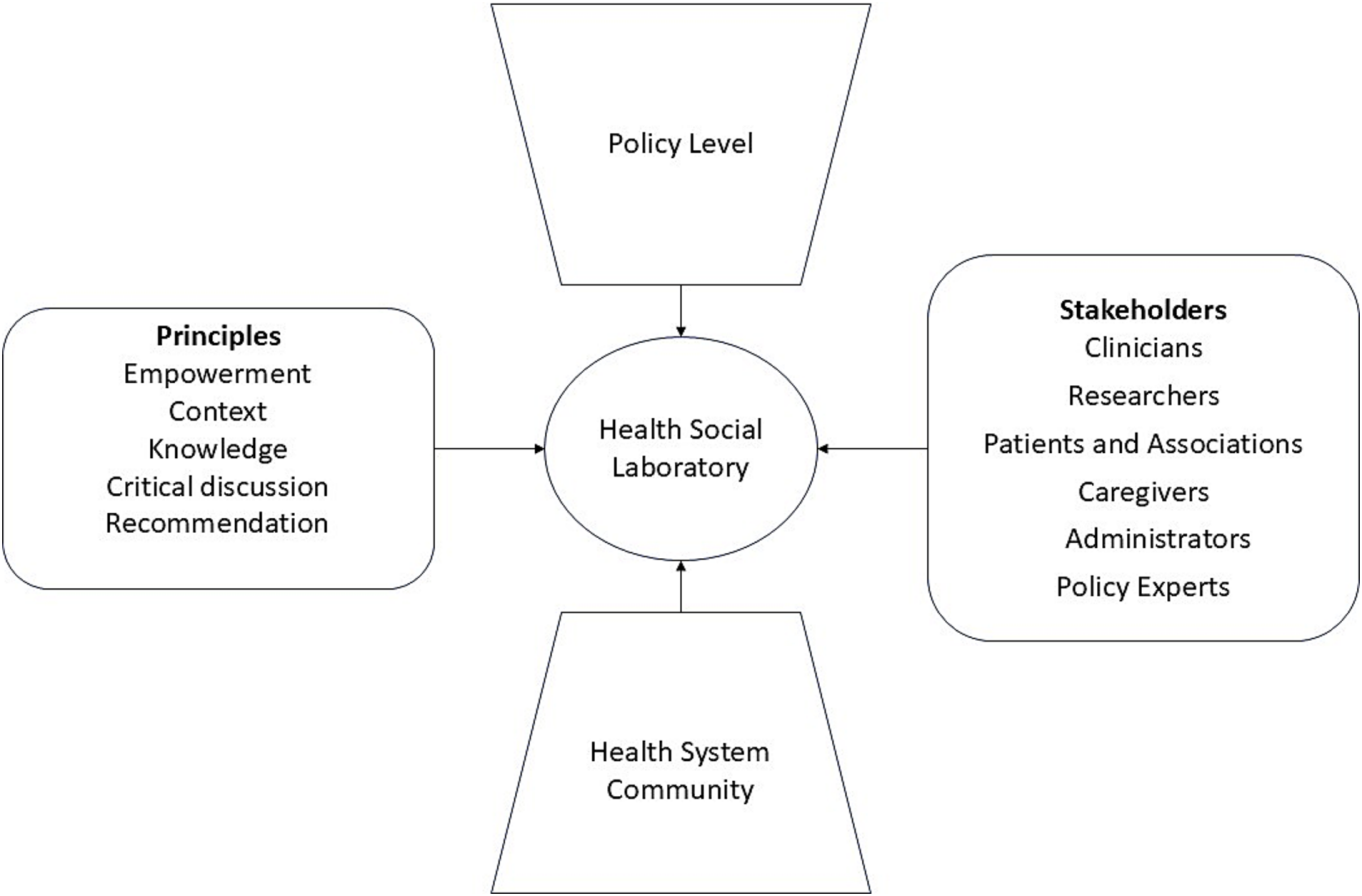
Conceptual framework chart for HSL design.

These elements and other principles of stakeholder engagement will be taken into account in the design of the HSL methodology, as shown in Figure 1.

**Figure 2:**
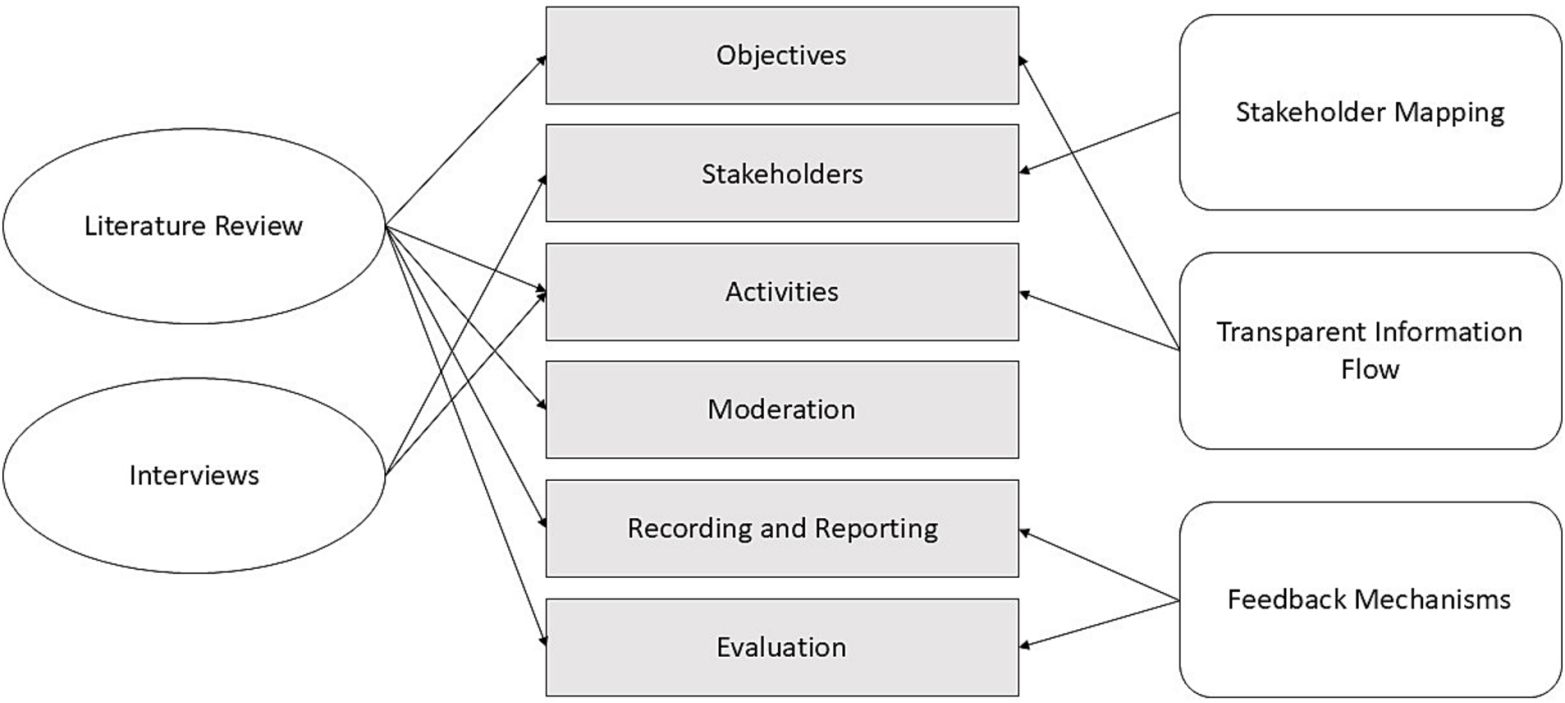
Links between study research and HSLs design.

The application of the above principles, combined with the literature review and interviews results, informed the design of HSLs, as depicted in Figure 2.

In particular, the two research types illustrated in this study helped us to identify features to create a meaningful collaboration space for Hereditary stakeholders.

Knowledge of obstacles and constraints to stakeholder participation gained from the literature review will be applied to the design of the lab structure and content, paying attention to the objectives, duration, moderation, and evaluation of activities.

Thanks to the interviews, instead, it was possible to identify the stakeholders that should be involved in HSLs. These include: clinicians or other health care professionals (e.g., nurses); health and technology researchers; patients and their representatives; caregivers or other patient relatives; health institutions administrators, that could be individuals responsible for managing the operations of healthcare facilities such as hospitals and/or clinics; and policy experts, representatives from local government, municipal or ministry entities, with knowledge of health-related policies or guidelines. Underrepresented groups will be included wherever possible, and special attention will be paid to ensuring gender inclusivity among participants.

The interviews also informed the choice of topics for the collection of stakeholders’ feedback. These will be technology, terminology and ethics. The reasoning is explained as follows: technology is going to integrate both the project goal of designing a technological environment for data analysis, and the need that was highlighted through the interviews to discuss interface, mediation, technology; terminology will consolidate the project efforts in the popularization of medical language, and allow stakeholders to discuss health communication strategies and approaches; ethics will cover the need for deliberation on the social aspects of research, favoring transparency, accessibility, and privacy.

In summary, the literature review and interview results allowed us to design a structured and detailed stakeholder engagement model involving the right set of stakeholders in discussion and decision-making to empower them, and using adequate discussion meetings well managed by a professional facilitator, to identify the problematic aspects and the potential for co-creating health initiatives and personalized communication.

## 5 Discussion

This study explored the main issues to address in developing Health Social Laboratories, an effective stakeholder collaborative relationship in the health information communication processes of the Hereditary project.

Firstly, through a literature review, we gathered that stakeholder engagement and participatory efforts in health research hold great potential: from the improvement of the long-term relevance of research, to the promotion of stakeholder trust (14, 15). We found, also, a number of challenges. Research shows both practical and theoretical constraints to effective participation, such as a lack of skills, time, or training on stakeholders’ or researchers’ side, or the risks of tokenistic and toxic engagement approaches, lacking meaningfulness and lasting impact (25). It is, thus, vital to encourage inclusive, early and sustained engagement, and to value stakeholders’ insight; always tailoring the participative methodology to the context-specific traits and challenges.

Secondly, through interviews with project partners, we could detect both a high multidisciplinarity and high variety of stakeholders within the consortium. Accordingly, different levels of public engagement, and significantly varied relationship networks must be considered in the HSLs. The interviews also permitted to recognize other challenges regarding health communication and technology. Customization and adaptation of health communication have been suggested, especially in terms of its complexity and technicality. Interviewees recognized data research requirements in terms of security and ethics, and the impact that this can have on patients’ perception of technology in clinical research and practice (26).

From this perspective, HSLs will develop an action research process by allowing the stakeholders involved to improve the level of communication in view of effective decision-making practices that in the health field have become more complex over time. Furthermore, the effort to connect various data sources, to actively involve different stakeholders, and particularly those less informed about the progress of health data collection and analysis systems will open up new possibilities for managing relationships between stakeholders, successfully fostering the co-creation of health technology communication systems.

Participatory practices are increasingly relevant in today’s society and communities as they can support a more ethical, specific and appropriate research. Furthermore, they can increase the likelihood of research results impact and implementation (28). In the case of HSLs, emphasis is placed on the possibility of focusing the discussion between stakeholders on technology and communication.

Considering critical aspects, systemic challenges can hinder the progress of these processes. For example, strict budgets and complex bureaucracy, past negative experiences with research that foster distrust, and funding mechanisms that focus on short-term results instead of long-term relationships, can greatly limit participatory research implementation (28). To effectively use HSLs in health contexts, the degree of influence that technologies can have must be taken into account, considering the progress and problems of data interpretation. In fact, the ability to manage large amounts of information or different types of data often creates difficulties for researchers, clinicians and patients. For this reason, HSLs must be accompanied by expert facilitators capable of harmonizing different skills and communication processes in an effective way.

In summation, with this paper we contribute to the design of HSLs: a flexible participative engagement model focused on improving health communication strategies in a technology-driven project. As seen in Figures 1, 2, HSLs design was ultimately informed by a multitude of principles and analysis efforts. This approach will allow the project stakeholders to take an active role in exploring, analyzing, and transforming health technology and communication issues under discussion. In fact, the laboratories are going to offer specific participative paths, which will encourage reflection and deliberation on themes of technology, terminology and ethics in health, effectively maximizing the value of stakeholders’ partnerships in the project.

Limitations of the study include the relatively small sample size of the interviews, which may not capture the full diversity of perspectives across stakeholders, and the specificity of the findings, related to the Hereditary project only, which may not be fully generalizable to other settings. In addition, the exclusion of patients from the interviews process was also a limit of the research. However, the participation of patients, patients’ representatives and caregivers in the participatory laboratories will allow to include their perspective in possible future tailoring of the methodology.

Future research should strive to expand the size and diversity of study samples, both in geographical and thematical terms. Including more regions, communities, and a wider array of health conditions, would enhance the generalizability of the findings and help explore how diverse factors and contexts influence health stakeholder engagement.

This research offers valuable insights into effective health stakeholder engagement at a time of great technological expansion due to the widespread use of artificial intelligence applications. Here, participative processes can facilitate deeper dialog and a more effective mutual understanding, bringing together expertise, needs, information management and communication.

According to this principle, HSLs will align the technical and scientific worlds with principles of humanities disciplines, helping all actors of the research and treatment process to actively contribute in an effective communication context.

## Data Availability

The raw data supporting the conclusions of this article will be made available by the authors, without undue reservation.
